# Link prediction based on spectral analysis

**DOI:** 10.1371/journal.pone.0287385

**Published:** 2024-01-02

**Authors:** Chun Gui

**Affiliations:** 1 College of Mathematics and Computer Science, Northwest Minzu University, Lanzhou, China; 2 Key Laboratory of China’s Ethnic Languages and Information Technology of Ministry of Education, Northwest Minzu University, Lanzhou, China; Lodz University of Technology: Politechnika Lodzka, POLAND

## Abstract

Link prediction in complex network is an important issue in network science. Recently, various structure-based similarity methods have been proposed. Most of algorithms are used to analyze the topology of the network, and to judge whether there is any connection between nodes by calculating the similarity of two nodes. However, it is necessary to get the extra attribute information of the node in advance, which is very difficult. Compared to the difficulty in obtaining the attribute information of the node itself, the topology of the network is easy to obtain, and the structure of the network is an inherent attribute of the network and is more reliable. The proposed method measures kinds of similarity between nodes based on non-trivial eigenvectors of Laplacian Matrix of the network, such as Euclidean distance, Manhattan distance and Angular distance. Then the classical machine learning algorithm can be used for classification prediction (two classification in this case), so as to achieve the purpose of link prediction. Based on this process, a spectral analysis-based link prediction algorithm is proposed, and named it LPbSA (Link Prediction based on Spectral Analysis). The experimental results on seven real-world networks demonstrated that LPbSA has better performance on Accuracy, Precision, Receiver Operating Curve(ROC), area under the ROC curve(AUC), Precision and Recall curve(PR curve) and balanced F Score(F-score curve) evaluation metrics than other ten classic methods.

## Introduction

The purpose of link prediction is to detect the missing links or forecast the future links based on the existing properties and structural topologies of the observed networks [1]. Link prediction includes prediction of unknown links and future links [2], it has important theoretical and practical significance. We can identify the spurious links in network, extract the implicit information. Furthermore, it helps us to model and evaluate the evolution mechanisms of network [3]. So far, link prediction has great practical applications in many areas, e.g., drug repositioning in biological networks [4], discovering underground criminal groups in terrorist networks [2], uncovering the disease relationships [5], finding new friends in social networks [6, 7], recommending the favorite goods for customers in online shopping systems [8], and predicting the potential collaborators in citation networks [9].

One of major type of approaches for link prediction is learning-based method. Such as classification-based method [10, 11], matrix factorization-based method [12, 13] and probabilistic model-based method [14–16]. All these methods have good performance, but they are time-consuming in constructing training data set. Another major type of approaches is similarity metric-based method [2, 3 17, 18], which is a simple and mainstream approach for link prediction. The similarity metric-based methods regard as that if two nodes are similar in attributes or network structure attributes, then they will form links with high probability [2]. However, the similarity metric-based methods based on node attributes often oriented towards specific contexts, which limits the scope of application in various networks. Besides, node attributes are difficult to obtain as they are always hidden or confidential. In contrast, the topology of the network is readily available which has good general adaptability with low computational complexity. So the structure-based similarity methods have been widely explored. A variety of topologies are employed to achieve good accuracy of link prediction, such as node degree, node centrality, neighborhood, clustering coefficient, community as well as path, and so on. Structure topology methods are more general to measure pair nodes similarity in networks. In the literature, lots of efforts have been devoted to node similarities [19], which are the attributes of common edge between two nodes. In the [20] article, an effective method for improving local random walks has been proposed, which encourages random walks to move towards nodes with greater impact at each step. Therefore, the next node is selected based on the influence of the source node. It considers the nodes that interact with each other and considers the neighboring nodes that interact with each other during the process of randomly walking to the next step, and randomly walks towards the nodes that are affected by the source node. The method do not require obtaining additional information about nodes in the network, but only use information about the network topology to predict whether there is a link between nodes. The probability of common edge between node pairs in the same community is greater than node pairs in different communities. The community structure has an influence on link prediction. [21] proposed a novel Non-negative Matrix Factorization (NMF) based algorithm called Graph regularized nonnegative matrix tri-factorization (GNMTF) model, which incorporates the intrinsic geometrical properties of the network graph by manifold regularization. Some of similarity measures such as SimRank [22] can be employed to calculate the similarity of an edge between a pair of nodes by only considering the topological structure in contrast to text-based similarity measure that consider the node content for similarity computation [23–25].

The link prediction model based on machine learning not only utilizes the structural information of the network, but also utilizes the attribute information of nodes in the network. This type of algorithm transforms link prediction into supervised classification or regression prediction problems by extracting the attribute features of various matrices in the network, and then various classic machine learning algorithms can be used. The matrix of the network, such as adjacency matrix and Laplacian matrix, is easy to obtain, but the use of machine learning algorithms requires the attributes of nodes. For example, in a shopping network, nodes represent shoppers or customers. Most customer information is confidential, and only relevant information about customers’ shopping can be obtained on the website. Such information is very limited, and its role in classification and regression is also limited; In protein interaction networks, the properties of proteins themselves are even more difficult to obtain; Various social networks have almost zero node information. Without node information, machine learning algorithms cannot perform classification predictions. Therefore, it can be said that the difficulty of link prediction based on machine learning is focused on how to obtain node attribute. One of the most important work is to calculate the similarity between nodes for the research of complex network structure. The methods include Jaccard Index based method [26–28], Euclidean distance, Manhattan distance, Angular distance and so on. Jaccard Index computes the similarity between nodes with a common edge, it is 0 if the two nodes share no common edge. The disadvantage of this method is that it only focuses on node pairs with common edges and ignores node pairs without common edges. Manhattan distance and Euclidean distance depend on the length of the line segment connecting the two nodes. Angular distance is another commonly used metric, and measures similarity as the angle between two vectors. Spectral analysis can be used for spectral clustering by using the eigenvectors of matrices [29]. It is easy to get matrices such as Adjacency matrix, Degree matrix, Laplacian matrix based on the topological structure of network. Spectral analysis considers the non-trivial eigenvector of Laplacian matrix. Each component of the minimum non-trivial eigenvector of the Laplacian matrix corresponds to a node in one network. Therefore, the non-trivial eigenvector can exactly be the attribute of the corresponding node, and the similarity between the pair nodes can be obtained by calculating the distance of the non-trivial eigenvector. In spite of the current work of similarity methods, we used spectral analysis for link prediction for the first time and proposed a method named LPbSA (link prediction based on spectral analysis). In this manuscript, we focus on the structure-based similarity methods and pay special attention to the similarity of node pairs based on Laplacian matrix. Different from the traditional method that node attributes are employed for link prediction, we get edge attributes and use the classification prediction methods of machine learning to classify the edges according to their attributes. Since the actual networks are usually sparse networks, the resulting edge data sets are unbalanced data sets [30]. SMOTE(Synthetic minority over-sampling technique) is employed to equilibrate the unbalanced data sets, and classification and prediction are executed on balanced data sets.

The rest of the manuscript is organized as follows. The related work of spectral analysis knowledge and unbalanced data set are introduced in section Related work. I introduced baselines and metrics for experiment in section Baselines and metrics. The description and pseudo code of the proposed LPbSA is given in section Description of LPbSA algorithm. The experiment preparation such as experimental networks, preparation of data sets and the choice of classifier are introduced in section Experiment preparation. The experimental analysis are given in section Experimental results and analysis. The conclusion of the article is in section Conclusion.

## Related work

The meanings of the symbols used in manuscript are shown in Table 1.

**Table 1:** The commonly used symbols.

Parameters	Meanings	Parameters	Meanings
*N*	number of nodes in the network	*M*	number of edges in the network
*N*(*G*)	the node set of *G*	*E*(*G*)	the edge set of *G*
*A*	the adjacency matrix of *G*	*D*	the degree matrix of *G*
*L*(*G*)	the Laplacian matrix of *G*	Γ(*i*)	the neighbor set of node *i*
Γ(*j*)	the neighbor set of node *j*	*k*(*i*)	the degree of node *i*
*k*(*j*)	the degree of node *j*	(*A*^*n*^)_*ij*_	path size with length *n* between nodes *i* and *j*
λ_1_	maximum eigenvalue of matrix *A*	*β*	weight attenuation factor
*ϕ*	a parameter less than 1	*α*	an adjustable parameter
$l_{ij}^{+}$	the value of the element in the corresponding position in matrix *L*^+^	*L* ^+^	pseudo inverse of Laplacian matrix *L*
*L* ^+^	pseudo inverse of Laplacian matrix *L*	*q*_*i*_	initial resource distribution of node
*P*_*ij*_ = *a*_*ij*_/*k*_*i*_	probability of particle *i* will go to node *j* in the next step	$|paths_{ij}^{<l>}|$	the number of paths with length *l* between *i* and *j*
*π*_*ij*_(*t*)	probability that the particle exactly right walk from node *i* to node *j* at time *t* + 1	*q*_*ij*_	probability of particle *i* eventually walked to node *j*

### Spectral analysis

The main content of this manuscript is to show how spectral analysis is used to achieve link prediction in all kinds of network. Spectral analysis is one of methods based on the properties of the spectrum of the matrix. By far, the most used matrix in spectral analysis is the Laplacian. The components of the non-trivial eigenvector of the Laplacian matrix exactly correspond to each node of the network, so the change of representation induced by the eigenvector makes the node attributes of the initial data set much more evident. Spectral clustering is one of the most important methods for community detection. We innovatively use spectral analysis for link prediction. The graph used in this manuscript are simple, un-weighted and undirected. Let *G* = (*N*, *E*) be a graph with node set *N*(*G*) and edge set *E*(*G*). We set *n* = |*N*(*G*)| and *m* = |*E*(*G*)|. The adjacency matrix of network *G* is denoted by *A* whose element *a*_*ij*_ is defined as follows:

$$\begin{array}{c} a_{ij}=\{\begin{array}{c} 1,\;if\;<v_{i},v_{j}>\;is\;an\;edge\;of\;G\\ 0,\;\;\;otherwise \end{array} \end{array}$$ (1)

The degree matrix of network *G* is denoted by *D* whose element *d*_*ij*_ is defined as follows:

$$\begin{array}{c} d_{ij}=\{\begin{array}{c} deg(v_{i}),\;if\;i=j\\ 0,\;\;\;\;\;\;\;\;\;otherwise \end{array} \end{array}$$ (2)

where the degree *deg*(*v*_*i*_) of a node counts the number of times an edge terminates at that node. *D* is a *n* × *n* diagonal matrix.

The topology of a network with *n* nodes can be shown by a symmetric *n* × *n* Laplacian matrix. The Laplacian matrix *L*(*G*) of graph *G* can be calculated as follows:

$$\begin{array}{c} L(G)=D(G)-A(G) \end{array}$$ (3)

### Unbalanced data sets

Classification with unbalanced data sets was listed as one of the top ten challenging problem in the field of data mining in 2005 ICDM(International Conference on Data Mining series). Most of the real world networks are sparse. Table 2 shows full connection edge number and actual edge number of seven networks used in experiment. It can be seen the smallest unbalanced rate is 19:1. In general, standard classifiers are developed to maximum a global measure of accurate, which has nothing to do with the class distribution. Classification of unbalanced data sets usually leads to a preference for the majority class, but less attention is paid to the minority class [31]. As a result, the minority class produce more error-prone than the majority one, as a large proportion of errors are concentrated in the minority class [32]. When one of the classes is heavily overpowered by the other one, the binary class data set is said to be unbalanced. We call the one having fewer of the number of samples as the minority class and the other one having more of the number of samples as the majority class. In this case, standard classification algorithms usually show a tilt to the majority class.

**Table 2:** Full connection edge number and actual edge number of seven networks.

Networks	Node size	Full connection edge number	Actual edge number	Unbalanced rate
USAir	332	54946	2126	25:1
Politic Blogs	1222	746031	16714	44:1
NetScience	1589	71631	2742	77:1
PPI	2375	2819125	11693	240:1
Power Grid	4941	12204270	6954	1755:1
Router	5022	12607731	6258	2014:1
Celegans	297	43956	2148	19:1

**Fig 1:**
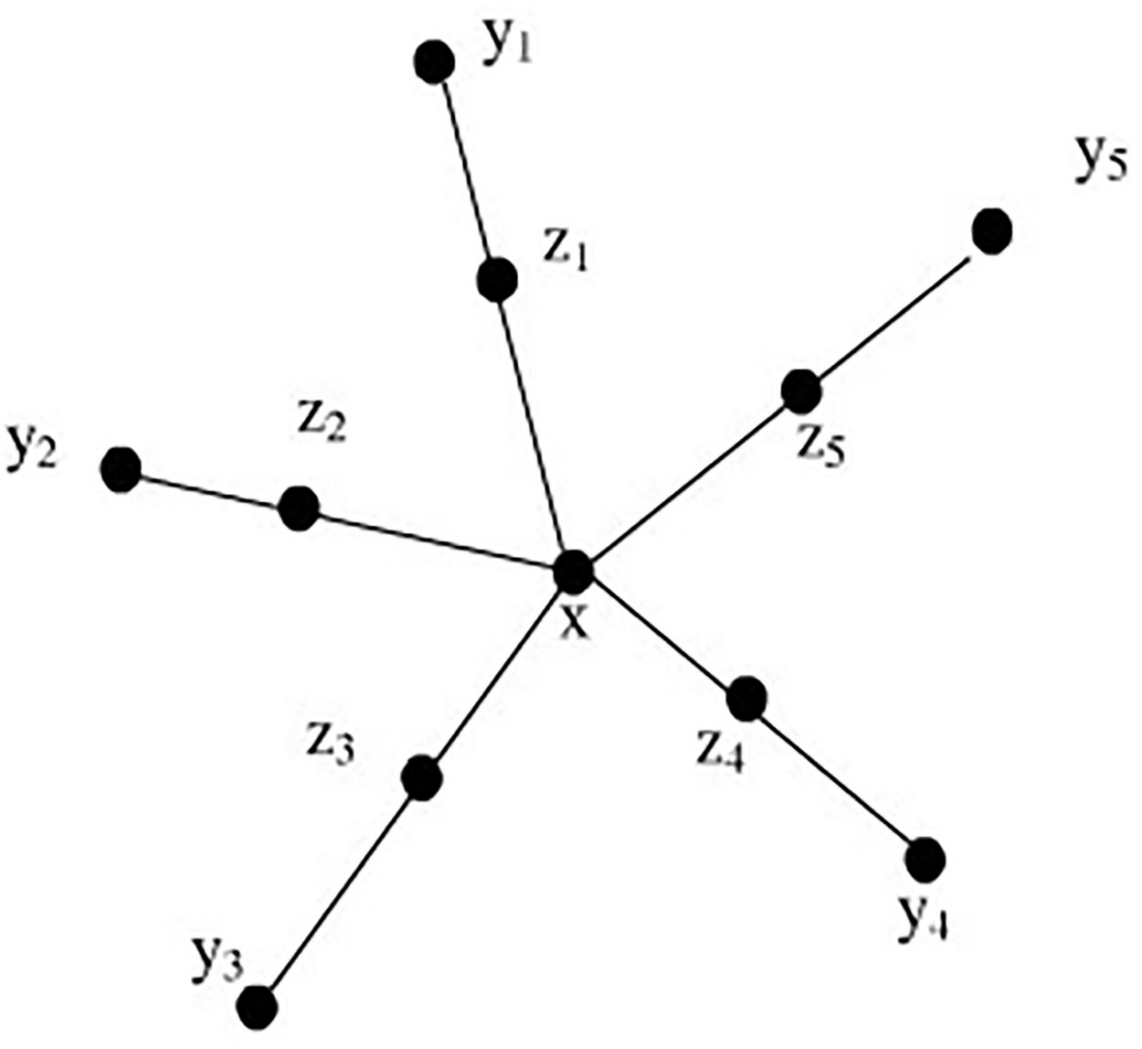
The sample composition of SMOTE [34].

A lot of methods are proposed in order to solve the problem of unbalanced data sets classification. These methods can be roughly divided into two categories. For unbalanced data sets, random undersampling Undersampling [33] and oversampling SMOTE(Synthetic Minority Over-Sampling Technique, SMOTE) [34] are two common data collection solutions. The sampling algorithm changes the category distribution of samples through a certain strategy to achieve the purpose of transforming unbalanced samples into relatively balanced samples. Undersampling randomly selects a small number of samples from the majority classes, and then combines the original minority samples as a new experimental data set. This method modifies the sample classification distribution by changing the sample proportion of majority classes. SMOTE algorithm first assumes that the samples between the close minority classes are still minority classes. Its idea is to synthesize new minority class samples. The synthesis strategy is to randomly select a sample *t* from its nearest neighbor for each minority class sample *s*, and then randomly select a point on the line between *s* and *t* as the newly synthesized “artificial” minority class sample, these three samples belong to the same category. As shown in Fig 1, which is the sample synthesis process of SMOTE method.

Let the sampling rate be *m*, for each minority sample *x*_*i*_, find out its *k* nearest neighbors, and select m nearest neighbors randomly *y*_*ij*_ (*j* = 1, 2, …, *m*), synthesize a new minority sample *z*_*j*_ (*j* = 1, 2, …, *m*) according to formula (4).

$$\begin{array}{c} z_{j}=x_{i}+rand\left(0,1\right)*\left(y_{ij}-x_{i}\right) \end{array}$$ (4)

where *rand*(0, 1) in the formula represents a random number between (0, 1).

## Baselines and metrics

### Comparison baselines

In order to evaluate the accuracy of the LPbSA method, we chose eighteen baselines for performance comparison based on six widely used metrics. In the following Eqs (5) to (23), *S*_*ij*_ represents the similarity of two nodes, Γ(*i*) represents the neighbor set of node *i*, Γ(*j*) represents the neighbor set of node *j*. *k*(*i*) means the degree of node *i*, *k*(*j*) means the degree of node *j*.

(1)CN [35, 36] (common neighbor index). This index counts the number of all common neighbors as similarity score and is defined as follows:

$$\begin{array}{c} S_{ij}^{CN}=|\Gamma \left(i\right)⋂\Gamma \left(j\right)| \end{array}$$ (5)

(2)Salton [37]. This index is the number of common neighbors of two nodes divided by the square root of the product of two nodes’ degree.

$$\begin{array}{c} S_{ij}=\frac{|\Gamma (i)⋂\Gamma (j)|}{\sqrt{k(i)\times k(j)}} \end{array}$$ (6)

(3)Jaccard [38]. This index is the sum of the number of common neighbors of two nodes divided by the number of all their neighbors.

$$\begin{array}{c} S_{ij}=\frac{|\Gamma (i)⋂\Gamma (j)|}{|\Gamma (i)⋃\Gamma (j)|} \end{array}$$ (7)

(4)Sorensen [39]. This index is the sum of twice the number of two nodes’ co-neighbors divided by the sum of two nodes’ degree.

$$\begin{array}{c} S_{ij}=\frac{2|\Gamma (i)⋂\Gamma (j)|}{k(i)+k(j)} \end{array}$$ (8)

(5)HPI [40] (hub promoted index). This index is the number of common neighbors of two nodes divided by the smaller degree of them.

$$\begin{array}{c} S_{ij}=\frac{|\Gamma (i)⋂\Gamma (j)|}{min\{k(i),k(j)\}} \end{array}$$ (9)

(6)HDI [41] (Leicht-Holme-Newman-I index). This index is the number of common neighbors of two nodes divided by the product of two nodes’ degree.

$$\begin{array}{c} S_{ij}=\frac{|\Gamma (i)⋂\Gamma (j)|}{max\{k(i),k(j)\}} \end{array}$$ (10)

(7) LHN-I [26] (Leicht-Holme-Newman-I index). This index is the number of common neighbors of two nodes divided by the product of two nodes’ degree.

$$\begin{array}{c} S_{ij}=\frac{|\Gamma (i)⋂\Gamma (j)|}{k(i)\times k(j)} \end{array}$$ (11)

(8)LHN-II [26] (Leicht-Holme-Newman-II index). This index takes into account all the paths between nodes, but gives different weights to different paths. Generally, short paths have a higher weight and longer paths have smaller weights. It is defined as follows:

$$\begin{array}{c} S=\beta A/E\left(A\right)+\beta^{2}A^{2}/E{\left(A\right)}^{2}+\beta^{3}A^{3}/E{\left(A\right)}^{3}+\ldots =M\lambda_{1}D^{-1}{\left(I-\phi A/\lambda_{1}\right)}^{-1}D^{-1} \end{array}$$ (12)

where *β* is weight the attenuation factor, *A* is the adjacency matrix of network, (*A*^*n*^)_*ij*_ represents the path size with length n between nodes *i* and *j*,*D* is the degree matrix, $E\left[{\left(A^{n}\right)}_{ij}\right]=\frac{k_{i}k_{j}}{M}\lambda_{1}^{n-1}$ is the expected value with length *n* between nodes *i* and *j*. *ϕ* is a parameter less than 1. λ_1_ is the maximum eigenvalue of matrix *A*.

(9)PA [42] (preferential attachment index). This index is the product of two nodes’ degree.

$$\begin{array}{c} S_{ij}=k\left(i\right)\times k\left(j\right) \end{array}$$ (13)

(10)AA [43] (Adamin-Adar index). This index is a variant of CN, which draws a distinction among common neighbors.

$$\begin{array}{c} S_{ij}=\Sigma_{z\in \Gamma (i)⋂\Gamma (j)}\frac{1}{logk(z)} \end{array}$$ (14)

where *k*(*z*) is the degree of node *z*.

(11)RA [41] (resource allocation index). Motivated by the resource allocation mechanism on networks, this index punishes the large degrees of common neighbors more heavily than AA.

$$\begin{array}{c} S_{ij}=\Sigma_{z\in \Gamma (i)⋂\Gamma (j)}\frac{1}{k(z)} \end{array}$$ (15)

(12)LP [44] (local path). This index only counts the number of paths with length 2 and 3 between two nodes and is defined as follows:

$$\begin{array}{c} S=A^{2}+\alpha A^{3} \end{array}$$ (16)

where *α* is a free parameter.

(13)LP* [41]. The result of LP is obtained at the optimal parameter *α*, and the result of LP* is obtained at a fixed parameter *α* = 0.01.

(14)Katz [45]. This index considers all paths between two nodes and assigns less weights to longer paths. It is defined as follows:

$$\begin{array}{c} S_{ij}=\Sigma_{l=1}^{\infty }\beta^{l}\cdot |paths_{ij}^{<l>}|=\beta A_{ij}+\beta^{2}{\left(A^{2}\right)}_{ij}+\beta^{3}{\left(A^{3}\right)}_{ij}+\cdots \end{array}$$ (17)

where *A* is the adjacency matrix of network and $|paths_{ij}^{<l>}|$ is the number of paths with length *l* between *i* and *j*. *β* is a tunable parameter that is always fixed at a very small value. If *β* is lower than the reciprocal of the maximum eigenvalue of adjacent matrix *A*, this index can be redefined as *S* = (*I* − *βA*)^−1^ − *I*.

(15)ACT [46] (average commute time). This index defines the similarity by calculating the average commute time between nodes, and the smaller the average commuting time for both nodes, the more similar they are. The numerical solution of the average commuting time can be obtained by solving the pseudo-inverse *L*^+^ of the Laplacian matrix corresponding to the network. It is defined as follows:

$$\begin{array}{c} t\left(i,j\right)=M\left(l_{ii}^{+}+l_{jj}^{+}-2l_{ij}^{+}\right) \end{array}$$ (18)

where $l_{ij}^{+}$ represents the value of the element in the corresponding position in matrix *L*^+^. *M* is the number of edges of the network. Based on the observed agglomeration effect of the network, the nearer the nodes are, the more likely they are to produce the connected edges. The similarity based on the average commuting time ACT is defined as follows:

$$\begin{array}{c} S_{ij}^{ACT}=\frac{1}{l_{ii}^{+}+l_{jj}^{+}-2l_{ij}^{+}} \end{array}$$ (19)

(16)RWR [47] (random walk with restart). RWR first assumes that random walked particles return to the initial node with a certain probability for each step taken. Based on this assumption, the probability vector of particle *i* arriving at other nodes at *t* + 1 moment is defined as follows:

$$\begin{array}{c} q_{i}\left(t+1\right)=cP^{T}q_{i}\left(t\right)+\left(1-c\right)e_{i} \end{array}$$ (20)

where *q*_*ij*_ is the probability of particle *i* eventually walked to node *j*. *P* is the Markov probability transfer matrix of the network. *P*_*ij*_ = *a*_*ij*_/*k*_*i*_ is the probability of particle *i* will go to node *j* in the next step. The element *a*_*ij*_ = 1 if there is an edge between nodes *i* and *j*, and 0 otherwise. (1 − *c*) is the probability of particle return. *e*_*i*_ is a one-dimensional vector and only the *i* − *th* element is 1, the rest of the elements are zero. The similarity of RWR is defined as follows:

$$\begin{array}{c} S_{ij}^{RWR}=q_{ij}+q_{ji} \end{array}$$ (21)

(17)LRW [48] (local random walk). LRW only care about the number of random walking steps. The similarity of LWR is defined as follows:

$$\begin{array}{c} S_{ij}^{LRW}=q_{i}\cdot \pi_{ij}\left(t\right)+q_{j}\cdot \pi_{ji}\left(t\right) \end{array}$$ (22)

where *π*_*ij*_(*t*) is the probability that the particle exactly right walk from node *i* to node *j* at time *t* + 1. *π*_*i*_(0) is a *N* × 1 dimensional vector and only the *i* − *th* element is 1, the rest of the elements are zero. *q*_*i*_ is the initial resource distribution of node.

(18)SRW [48] (superposed random walk). SRW is the sum of the *t* − *th* step of SRW and its previous results. The similarity of SRW is defined as follows:

$$\begin{array}{c} S_{ij}^{SRW}=\Sigma_{l=1}^{t}s_{ij}^{LRW}\left(l\right)=q_{i}\Sigma_{l=1}^{t}\pi_{ij}\left(l\right)+q_{j}\Sigma_{l=1}^{t}\pi_{ji}\left(l\right) \end{array}$$ (23)

The meaning of the parameter is the same as that of Eq 22.

### Evaluation metrics

The operation object of link prediction is the network that can be transformed into graph. Consider a simple network *G*(*V*, *E*) be a graph with vertex set *V* and edge set *E*, let *U* denotes all possible edges of *G* and includes *E*. In order to test the accuracy of predictors, all possible edges *U* are randomly divided into two parts: one part is training set *U*^*T*^ and the other part is testing set *U*^*P*^. Link prediction is to predict the possibility of generating edges between two nodes through known network structure information. *U*^*T*^ is regarded as the foregone information of network, while *U*^*P*^ is used to validate the accuracy of classifier and does not used to participate in the prediction procedure. The set of edges for machine learning classification prediction has the following relation: *U*^*T*^ ⋃ *U*^*P*^ = *U* = *n* × (*n* − 1)/2 and *U*^*T*^ ⋂ *U*^*P*^ = *ϕ*, where *n* is the node size of *G*. Seven metrics such as Accuracy, Precision, Recall, AUC, ROC curve, PR curve and F-Score are used to measure the prediction accuracy. Confusion matrix [49] is a specific table layout which allows visualization of the performance of algorithm as show in Table 3.

**Table 3:** Confusion matrix of four terms of measure.

	Prediction outcome		Total
Actual value	TP	FP	P
	FN	TN	N
Total	P’	N’	

*TP* means true positive, which refers to the positive case that is correctly classified by the model. *TN* means true negative, which refers to the negative case that is correctly classified by the model. *FP* means false positive, which refers to the negative case that is incorrectly classified as positive by the model. *FN* means false negative, which refers to the positive case that is incorrectly classified as negative by the model. The evaluation metrics are described as follows:

$$\begin{array}{c} Accuracy=\frac{TP+TN}{TP+TN+FP+FN} \end{array}$$ (24)

$$\begin{array}{c} Precision=\frac{TP}{TP+FP} \end{array}$$ (25)

$$\begin{array}{c} AUC=\int_{0}^{1}TPRd_{FPR}=\frac{1}{\left(TP+FN)(TN+FP\right)}\int_{0}^{1}TPd_{FP} \end{array}$$ (26)

$$\begin{array}{c} Recall=\frac{TP}{TP+FN} \end{array}$$ (27)

$$\begin{array}{c} F-Score=\frac{Precision*Recall*2}{Precisi+Recall} \end{array}$$ (28)

AUC can directly see the performance of the classifier through an accurate value. AUC represents the area under the ROC. The AUC value range is between 0 and 1. The larger the AUC value, the higher the accuracy of the algorithm. Therefore, the ideal value of AUC is 1. However, such an ideal classifier does not exist. Generally, when the AUC value is greater than 0.5, it means that the classification result of the classifier is better than the random classification result. In addition to these five numerical evaluation indicators, we used ROC(Receiver Operating Curve), PR(Precision and Recall curve) and F-Score curve to show the results of classification prediction in a two-dimensional space by graphical way. ROC is a method to display classification prediction results in a two-dimensional space. The abscissa is FPR(False Positive Rate), and the ordinate is TPR(True Positive Rate), where FPR = FP/(TN+FP) is the proportion of positive samples which are incorrectly divided, TPR = TP/(TP+FN) is the proportion of positive samples which are correctly divided, and the coordinates of ROC curve (0, 1) indicate that all samples are correctly divided. ROC does not have an accurate value to reflect the accuracy, so it cannot directly reflect the performance of the classifier. In PR curve, *P* represents precision and *R* represents recall. It represents the relationship between accuracy and recall. Generally, recall is set as abscissa and precision as ordinate.

### The similarity definition between nodes

It is necessary to calculate the similarity between each node pairs for link prediction. We measure three kinds of similarity based on non-trivial eigenvectors of Laplacian Matrix of network, such as Euclidean distance, Manhattan distance and Angular distance. All these distance are the attributes of common edge. Given the two data points *A* = (*a*_1_, *a*_2_, …, *a*_*n*_) and *B* = (*b*_1_, *b*_2_, …, *b*_*n*_), the Euclidean distance is defined as:

$$\begin{array}{c} d_{AB}^{E}=\sum_{k=1}^{n}\sqrt{{\left(a_{k}-b_{k}\right)}^{2}} \end{array}$$ (29)

the Manhattan distance of the two data points is defined as:

$$\begin{array}{c} d_{AB}^{M}=\sum_{k=1}^{n}|\left(a_{k}-b_{k}\right)| \end{array}$$ (30)

the cosine value of the two data points is defined by formula:

$$\begin{array}{c} cos\left(\vec{A},\vec{B}\right)=\frac{\vec{a_{k}}•\vec{b_{k}}}{\|\vec{a_{k}}\left\|\right\|\vec{b_{k}}\|} \end{array}$$ (31)

in practical application, the inverse cosine is used to implement the comparison between objects instead of cosine value. The Angular distance of the two data points is defined as:

$$\begin{array}{c} d_{AB}^{A}=arccos\frac{\vec{a_{k}}•\vec{b_{k}}}{\|\vec{a_{k}}\left\|\right\|\vec{b_{k}}\|} \end{array}$$ (32)

Manhattan distance and Euclidean distance depend on the length of the line segment connecting the two nodes(called absolute values). Angular distance is a commonly used metric which measures similarity as the angle between two vectors. These three methods have their own advantages and disadvantages. They can learn from each other to make the acquired attribute values more comprehensive.

## Description of LPbSA algorithm

With the similarity calculation based on Laplacian matrix is introduced, spectral analysis is employed for link prediction. We named this method as LPbSA. The pseudocode of LPbSA is shown in Table 4. To clarify the steps of the algorithm, I select a small network to demonstrate the results of the algorithm step by step. You can see the detailed steps in S1 Appendix.

**Table 4:** Pseudocode of LPbSA.

**Input**: Network *G*
1: *p* ← number of nodes in *G* 2: *D* ← degree matrix of *G* 3: *A* ← adjacency matrix of *G* 4: *Laplacian*(*G*) ← *D* − *A* 5: *vec*1, *vec*2,*vec*3 ← the minimum three nontrivial eigenvectors of *Laplacian*(*G*) 6: **for** *i* in 1 : *p*, **do** 7: **for** *j* in 1 : *p*, **do** 8: *a*. *Attr*1,*Attr*2,*Attr*3 ← Euclidean Distance, Manhattan Distance and Angular Distance of *vec*1 and *vec*2 9: *b*. *Attr*4,*Attr*5,*Attr*6 ← Euclidean Distance, Manhattan Distance and Angular Distance of *vec*1, *vec*2 and *vec*3 10: **endfor** 11: **endfor** 12: *cla*=[] 13: **for** *i* in 1 : *p*, **do** 14: **for** *j* in (*i* + 1) : *p*, **do** 15: *cla*=*cla*+A[i][j] 16: **endfor** 17: **endfor** 18: combine *attr*1, *attr*2, *attr*3, *attr*4, *attr*5, *attr*6 and *cla* as a *newdataset* 19: use SMOTE method to get the *balanced* *data* *set* 20: use RF for the classification prediction on the *balanced* *data* *set*
Output: the classification results of the *balanced* *data* *set*

**Note**: *attr*1, *attr*2, *attr*3, *attr*4, *attr*5, *attr*6 are the six columns of attribute and *cla* is the column of classification value of new data set

## Experiment preparation

### Experimental networks

There are seven real world networks from various fields with different structures are considered in experiment. The basic topological attributes of networks are given in Table 5. In our experiments, the giant component of each network is only considered. The descriptions of these seven networks are as follows: (1) USAir [50] : a network records the routes of a certain period of the United States; (2) Politic Blogs [51]: an US political blog network; (3) NetScience [52]: a network of scientists on network theory and experiment; (4) PPI [53]: a protein-protein interaction network of yeast; (5) Power Grid [54]: a power grid network of the western US; (6) Router [55]: a snapshot of the Internet autonomous system; (7) Celegans [56]: a neural network of the nematode Caenorhabditis elegans.

**Table 5:** The topology attributes of seven networks.

Network	*N*	*M*	*LCS*	*C*	*MC*	*H*	<k>
USAir	332	2126	332/1	0.749	-0.208	3.46	12.80723
Politic Blogs	1224	19090	1222/2	0.361	-0.079	3.13	27.35516
NetScience	1589	2742	379/268	0.878	0.462	1.85	4.823219
PPI	2617	11855	2375/92	0.387	0.461	3.73	9.846737
Power Grid	4941	6954	4941/1	0.107	0.003	1.45	2.669095
Router	5022	6258	5022/1	0.033	-0.138	5.05	2.492234
Celegans	297	2148	297/1	0.308	-0.163	1.801	15.88552

**Note**: *N* is the node size. *M* is the link size. *LCS* is the largest connected subset. *C* is the average clustering coefficient. *MC* is the matching coefficient. *H* is the network heterogeneity. <*k*> is the average degree of nodes.

### Preparation of data set

SMOTE method is used to balance the number of edges of the data set. SMOTE is proposed in order to solve the shortage of random sampling. It combines the newly synthesized minority sample with the sampled majority sample to obtain a relatively balanced new data set, which lays a foundation for the bisectional modeling of unbalanced data set. There is no definitive conclusion as to how much the unbalanced rate can achieve good classification results [57]. Table 6 is the parameter selection for seven data sets. Specific explanation on the two parameters is as follows: assume that the original data set consist of *N* minority samples and *M* majority samples, two parameters *perc*.*over* = *a*, *perc*.*under* = *b*. First, increase the size of minority samples, about *a*/100 new samples are added to each sample, there are a total of a*N/100 new minority class samples are added. Put the original minority class sample and the new minority class sample into the new data set. Then sample the majority sample, the sample size is (b/100) * a * N/100, get a new majority sample, put the new majority sample into the new data set. The minority size sample is (1 + *a*/100) * *N*, and the majority size sample is (b/100) * a * N/100. The two parameter values perc.over and perc.under are obtained through experiments over and over again. The purpose of using these two parameters is to obtain a balanced data set using the SMOTE method. Due to the different imbalance rates of each data set, there is no unified parameter setting method to obtain a balanced data set. Table 7 shows the number of edges that the network contains before and after the SMOTE method is used.

**Table 6:** The value of parameter.

Network	Celegans	PPI	NetScience	PowerGrid	Politic Blogs	Router	USAir
perc.over	150	40	150	180	15	200	150
perc.under	240	350	200	200	400	150	200

**Table 7:** The size of edge before and after SMOTE is used.

Network	Celegans	PPI	NetScience	PowerGrid	Politic Blogs	Router	USAir
B-Majority	41808	2807432	70717	12197676	729317	12601473	52820
B-Minority	2148	11693	914	6594	16714	6258	2126
A-Majority	5155	16369	1828	13188	17549	18774	4252
A-Minority	4296	16370	1828	13188	19221	18774	4252

Note: B-Minority is the number of minority sample before SMOTE is used; B-Majority is the number of majority sample before SMOTE is used; A-Minority is the number of minority sample after SMOTE is used; A-Majority is the number of majority sample after SMOTE is used.

The balanced data sets are the experimental data sets, which are divided into training set and testing set. The training set occupies 70% and the testing set occupies 30%. We use *R* language to carry out experiment. The Random Forest algorithm uses the *RF* package of *R*, in which the number of growing trees is set as 100, and the ratio of training set to testing set is 7 : 3. The hardware environment is Intel (R) Core i7–4790 CPU @ 3.60GHz, memory is 8G, operating system is Microsoft Windows 7 64-bit. We finished the whole experiments by using free software named *RStudio*. The methodology is implemented in *RStudio* freely available for the interested users. First we download *R*3.4.1 from URL https://www.r-project.org/.RStudio is an active member of the *R* community, which makes *R* easier to use. It includes a code editor, debugging and visualization. We draw all graphs in this manufacture based on *RStudio*. In order to get more accurate experimental results, each experiment of data set is repeated 20 times.

### The choice of classifier

The data sets processed by Undersampling and SMOTE should be classified and predicted. The experiment compares the prediction results of five popular classifiers: Random Forest(abbreviated as RF) [58], Decision Tree(abbreviated as DT) [59, 60], K-nearest neighbor(abbreviated as KNN) [61], Support Vector Machine(abbreviated as SVM) [62, 63] and Neural Network(abbreviated as nnet) [64, 65]. Table 8 shows the results of Accuracy, Precision, AUC, Recall and F-score values obtained from RF classification prediction. For each evaluation index, the data sets processed by SMOTE method get better results than the ones processed by Undersampling. Because the Undersampling method discards some samples of the original data set, resulting in the loss of valuable information, while the SMOTE method makes full use of the information of a few classes in the original data set. SMOTE is based on the *k* nearest neighbor sample points of each sample point, randomly selecting *N* neighboring points to multiply the difference by a threshold within the range of [0, 1], in order to achieve the purpose of synthesizing data. The core of this algorithm is that the features of adjacent points in the feature space are similar. It does not sample in the data space, but in the feature space, so its accuracy is higher than traditional sampling methods.

**Table 8:** The results of two balance methods use RF classifier to measure the prediction accuracy on seven data sets using five evaluation indexes.

Balance method and evaluation indexes		Celegans	PPI	NetScience	Power Grid	Politic Blogs	Router	USAir
Undersampling	Accuracy	0.759	0.961	0.933	0.975	0.838	0.943	0.832
	Precision	0.73	0.901	0.922	0.966	0.842	0.934	0.803
	AUC	0.825	0.961	0.977	0.995	0.894	0.988	0.911
	Recall	0.818	0.892	0.946	0.985	0.901	0.952	0.881
	F-Score	0.773	0.896	0.934	0.975	0.871	0.943	0.840
SMOTE	Accuracy	0.806	0.911	0.965	0.980	0.838	0.956	0.930
	Precision	0.839	0.927	0.952	0.976	0.842	0.956	0.909
	AUC	0.943	0.968	0.992	0.996	0.894	0.990	0.981
	Recall	0.921	0.934	0.980	0.992	0.901	0.976	0.957
	F-Score	0.878	0.931	0.965	0.984	0.871	0.966	0.932

**Fig 2:**
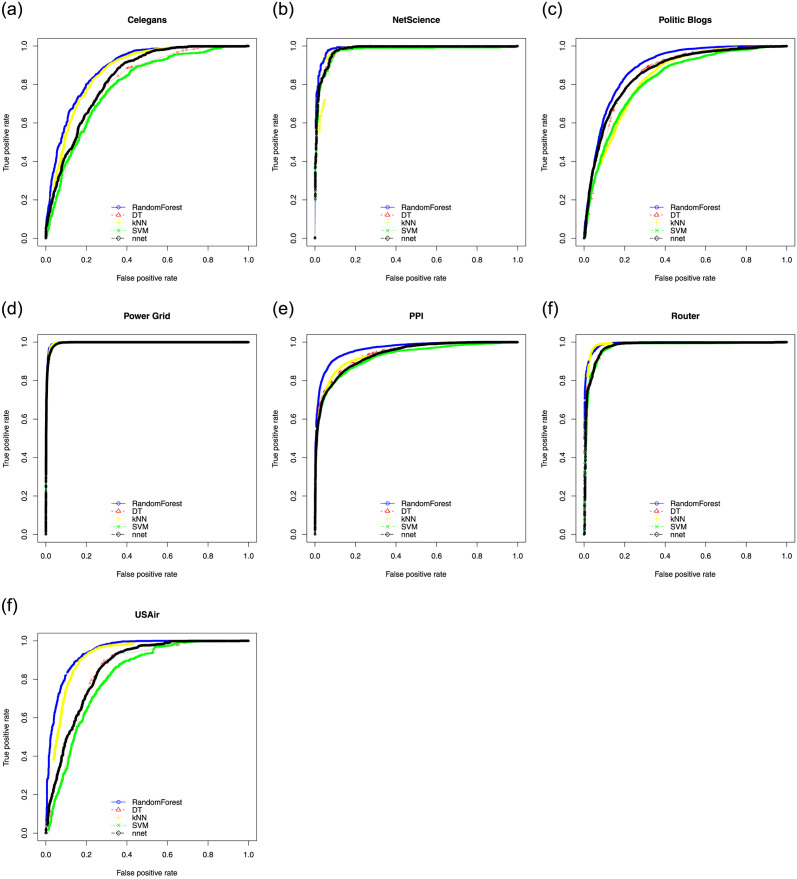
ROC curves of seven data sets used SMOTE((a) for Celegans network, (b) for NetScience network, (c) for Politic Blogs network, (d) for Power Grid network, (e) for PPI network, (f) for Router network and (g) for USAir network).

**Fig 3:**
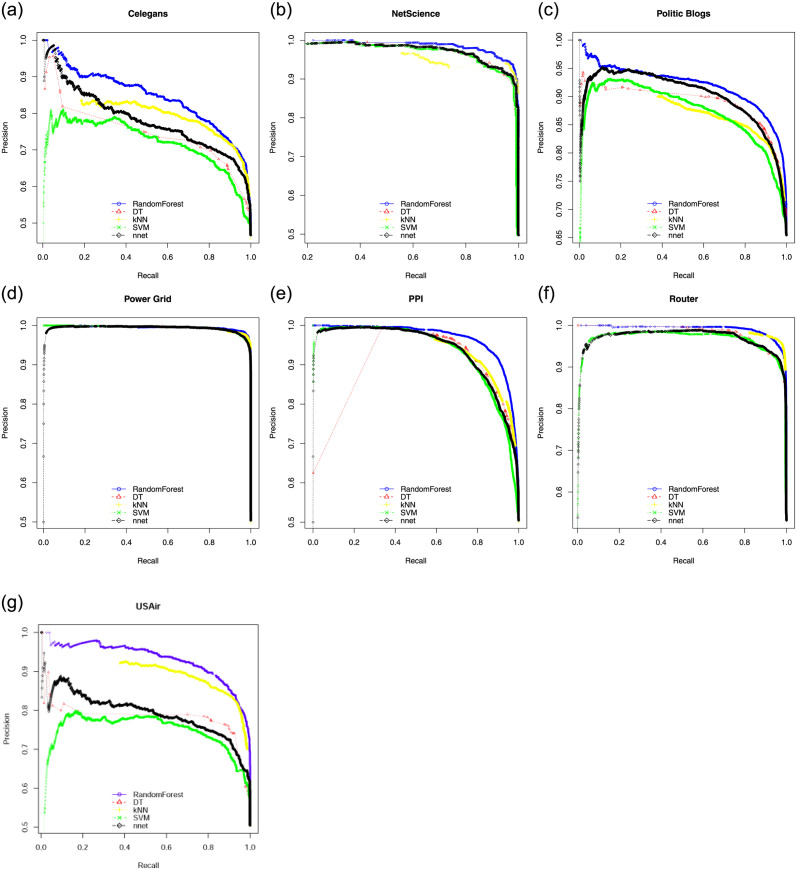
PR curves of seven data sets used SMOTE((a) for Celegans network, (b) for NetScience network, (c) for Politic Blogs network, (d) for Power Grid network, (e) for PPI network, (f) for Router network and (g) for USAir network).

**Fig 4:**
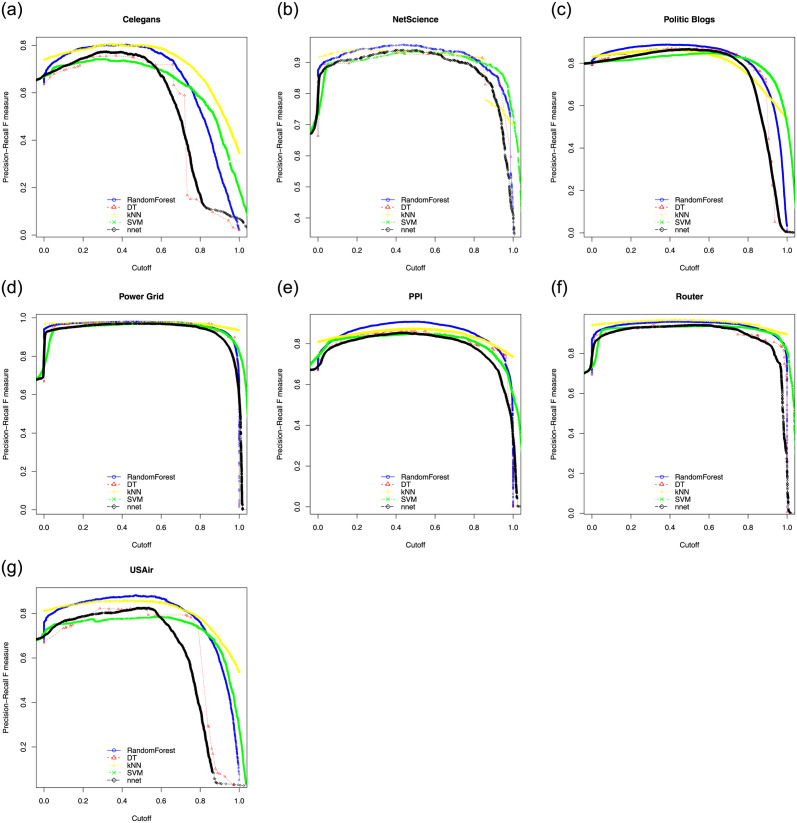
F-Score curves of seven data sets used SMOTE((a) for Celegans network, (b) for NetScience network, (c) for Politic Blogs network, (d) for Power Grid network, (e) for PPI network, (f) for Router network and (g) for USAir network).

Here we show ROC, PR curves and F-Score curves of these five classifiers on seven experimental data sets. In general, if the curve is smooth, it means there is not much over-fitting. For ROC, the closer the curve is to the upper left corner in the coordinate system, the better. For PR curves and F-Score curves, the closer the curve is to the upper right corner in the coordinate system, the better. Fig 2 consists of the ROC of five classifiers. Fig 3 consists of the PR curves of five classifiers. Fig 4 consists of the F-Score curves of five classifiers.

From these three sets of curves, we can see that in the ROC curves, RF is only slightly inferior to the KNN model on the Router data set, but obtains the best performances on the other six data sets. In the PR curves, RF obtains the best performances on all seven data sets. In the F-Score curves, RF, SVM and KNN models have their own advantages and disadvantages. With the comprehensive evaluation, RF is the best one among five classifiers. RF adopts the bagging idea, it does not need a single decision tree to make prediction, but votes and selects the classification results of multiple weak classifiers. In this process, multiple weak classifiers form a strong classifier. Therefore, RF under the bagging idea improves the prediction accuracy.

## Experimental results and analysis

### Experimental results

We use Accuracy, Precision, Recall, AUC, ROC, PR curve and F-Score metrics to measure the performance of prediction. Tables 9–11 respectively show the accuracy results measured by Accuracy, Precision and AUC on seven networks compare with other ten methods.

**Table 9:** Prediction accuracy measured by Accuracy on six networks.

Method	PPI	NetScience	Power Grid	Politic Blogs	Router	USAir
LPbSA	***0.911***	***0.965***	***0.980***	0.838	***0.956***	0.930
CN	0.889	0.933	0.590	0.925	0.559	0.937
Salton	0.869	0.911	0.585	0.874	0.552	0.898
Jaccard	0.888	0.933	0.590	0.882	0.559	0.901
Sorensen	0.888	0.933	0.290	0.881	0.559	0.902
HPI	0.868	0.911	0.585	0.852	0.552	0.857
HDI	0.888	0.933	0.590	0.877	0.559	0.895
LHN-I	0.866	0.911	0.585	0.772	0.552	0.758
PA	0.828	0.623	0.446	0.907	0.464	0.886
AA	0.888	0.932	0.590	0.922	0.559	0.925
RA	0.890	0.933	0.590	***0.931***	0.559	***0.955***

The bold and oblique values are the best ones.

**Table 10:** Prediction accuracy measured by Precision on seven networks.

Method	Celegans	PPI	NetScience	Power Grid	Politic Blogs	Router	USAir
LPbSA	***0.839***	***0.927***	***0.952***	***0.976***	***0.842***	***0.956***	***0.909***
LP	/	0.734	0.292	0.132	0.519	0.557	0.627
LP*	/	0.734	0.292	0.132	0.469	0.121	0.627
Katz	/	0.719	0.290	0.063	0.456	0.368	0.623
LHN-II	/	0	0.060	0.005	0	0	0.005
ACT	0.07	0.57	0.19	0.08	/	/	0.49
RWR	0.13	0.52	0.55	0.09	/	/	0.65
LRW	0.14	0.86	0.54	0.08	/	/	0.64
SRW	0.14	0.73	0.54	0.11	/	/	0.67

The bold and oblique values are the best ones.

**Table 11:** Prediction accuracy measured by AUC on seven networks.

Method	Celegans	PPI	NetScience	Power Grid	Politic Blogs	Router	USAir
LPbSA	***0.943***	0.968	***0.992***	***0.996***	0.894	***0.990***	***0.981***
LP	/	0.970	0.988	0.697	0.941	0.943	0.960
LP*	/	0.970	0.988	0.697	***0.939***	0.941	0.959
Katz	/	0.972	0.988	0.952	0.936	0.975	0.956
LHN-II	/	0.968	0.986	0.947	0.769	0.959	0.778
ACT	0.747	0.900	0.934	0.895	/	/	0.901
RWR	0.889	0.978	0.993	0.760	/	/	0.977
LRW	0.899	0.974	0.989	0.953	/	/	0.972
SRW	0.906	***0.980***	***0.992***	0.963	/	/	0.978

The bold and oblique values are the best ones.

**Fig 5:**
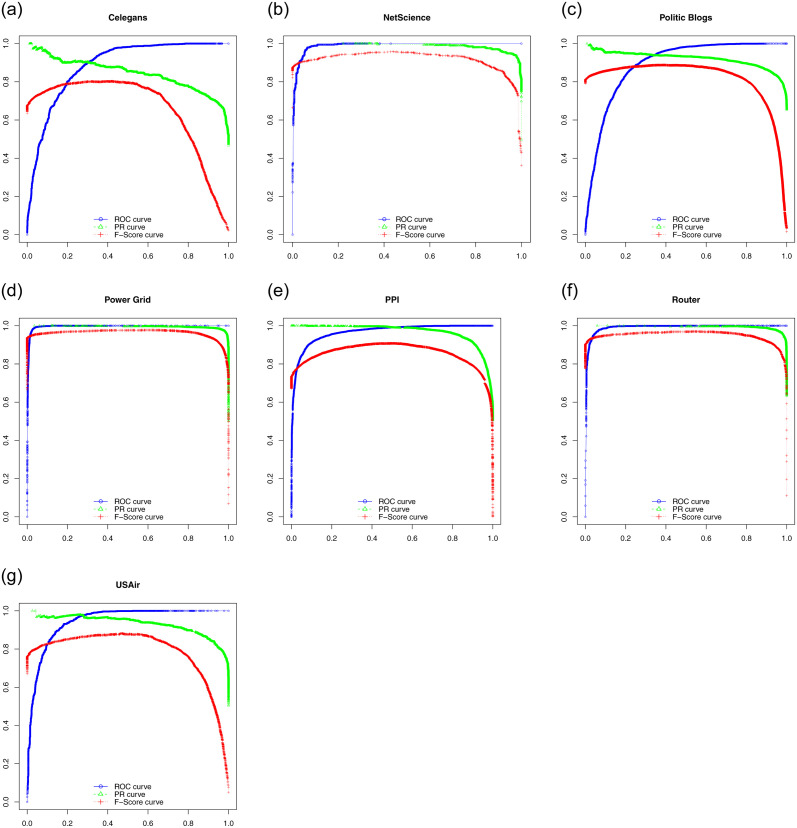
Prediction performance of LPbSA measured by ROC, PR curves and F-Score curves((a) for Celegans network, (b) for NetScience network, (c) for Politic Blogs network, (d) for Power Grid network, (e) for PPI network, (f) for Router network and (g) for USAir network).

Besides these three evaluation metrics, we use ROC, PR curves and F-Score curves to show the prediction performance of LPbSA by a graphical way. Fig 5 shows the ROC, PR curves and F-Score curves of LPbSA on seven networks.

### Experimental analysis

#### Analysis of experimental results

The prediction accuracy measured by Accuracy in Table 7, LPbSA is not ideal on Politic Blogs data set with value 0.838 and a little inferior to CN and RA on USAir data set with value 0.930, but obtains good performance on other four data sets. The prediction accuracy measured by Precision in Table 8, LPbSA gets the best prediction accuracy on seven data sets. The prediction accuracy measured by AUC in Table 9, LPbSA is slightly poorer on PPI data set with value 0.968, and is superior to ACT with value 0.900 and equal to LHN-II with value 0.968, but obtains good performance on other six data sets. With the comprehensive evaluation, we think LPbSA is the better one compare to other compared methods. From Fig 5 we can see: 1.most curves are smooth; 2.ROC curves are closer to the upper left corner except Celegans and Politic Blogs data sets; 3.PR and F-Score curves are closer to the upper right corner except Celegans data set. The performances of LPbSA are good at most data sets.

The superiority of the proposed method are: a. each component of the minimum non-trivial eigenvector of the Laplacian matrix corresponds to a node in one network. The distance based spectral analysis exactly reflects the similarity between pair nodes; b. the real networks are usually sparse, the resulting data sets are unbalance. SMOTE is employed to improve the category distribution of samples to get the balanced data sets. c. RF adopts the bagging idea, it does not need a single decision tree to make prediction, but votes and selects the classification results of multiple weak classifiers. In this process, multiple weak classifiers form a strong classifier. Therefore, RF under the bagging idea improves the prediction accuracy. The experimental results also prove that RF performs the best among the five classifiers. Therefore, the proposed method uses RF to complete classification prediction. Compared to the baseline methods, the proposed method uses more precise attributes to complete classification predictions on balanced datasets, better link prediction performance was achieved.

#### Analysis of algorithm complexity

Let *n* and *m* are the number of nodes and edges of the network, respectively. The first computationally expensive part of LPbSA algorithm is the calculation of Laplacian eigenvectors, which is *O*(*n*^3^). The Lanczos method can be used to determine the required eigenvectors [62]. Thus the computational complexity is reduced to *n*/λ_3_ − λ_2_, where λ_3_ is the second non-trivial eigenvalue and λ_2_ is the first non-trivial eigenvalue. The second computationally expensive part of LPbSA is using RF for classification prediction. The complexity of RF is *O*(*nklogn*), where *k* = (*the number of column of balanced data set* − 1), *k* = 5 in the proposed method. The calculation of this algorithm is mainly spent on obtaining the eigenvectors of the Laplacian matrix, so the complexity of LPbSA is *n*/λ_3_ − λ_2_.

## Conclusion

In the present research, spectral analysis is used for link prediction for the first time, which considers the non-trivial eigenvector of Laplacian matrix. Each component of the minimum non-trivial eigenvector of the Laplacian matrix corresponds to a node in one network. Therefore, the non-trivial eigenvector can exactly be the attribute of the corresponding node, and the similarity between the pair nodes can be obtained by calculating the distance of the non-trivial eigenvector. Different from the traditional method that node attributes are employed for link prediction, we get edge attributes and use the classification prediction methods of machine learning to classify the edges according to their attributes. In this process, since the actual networks are usually sparse networks, the resulting edge data sets are unbalanced data sets. So, SMOTE is employed to equilibrate the unbalanced data sets, and RF based classification and prediction are executed on balanced data sets. In order to prove the performance of the proposed method, a comparative experiment was performed on seven real-world networks. It demonstrated that LPbSA has better performance on Accuracy, Precision, AUC, ROC curve, PR curve and F-score curve evaluation metrics than other ten classic methods. In future studies, the proposed method will have the option to be applied to weighted and directed networks. The future study will be link prediction based spectral analysis on large-scale networks through distributed computing.

## Supporting information

S1 Appendix (PDF)
