# Correction: Link prediction based on spectral analysis

**DOI:** 10.1371/journal.pone.0298926

**Published:** 2024-02-13

**Authors:** The PLOS One Staff

There are errors in the Funding section. The correct Funding statement is: This work is partly supported by the Fundamental Research Funds for the Central Universities (NO:31920230171).

The publisher apologizes for the error.
