# Supplementary material for: Link prediction based on spectral analysis
Source: PLoS One. 2024 Jan 2;19(1):e0287385. doi: 10.1371/journal.pone.0287385 (PMC10760775; doi:10.1371/journal.pone.0287385)
Supplement: S1 Appendix — (PDF) [file pone.0287385.s001.pdf]

Appendix 1

To clarify the steps of the algorithm, we selected a small network to demonstrate the results of the algorithm step by step. This small network is consist of 9 vertices and 13 edges which is come from the article(Y.-Y. Ahn, J. P. Bagrow, S. Lehmann, Link communities reveal multiscale complexity in networks, Nature 466 (7307) (2010) 761–764. doi:10.1038/ nature09182). Its topology is shown in Fig.1. It is consist of 9 vertices and 13 edges.

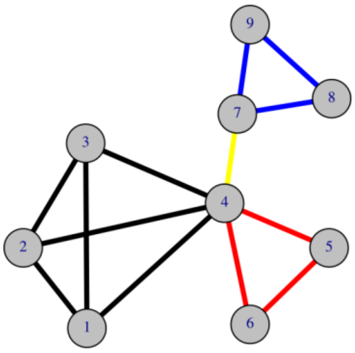

Fig.1 the topology of the small network

Step 1: Obtain the adjacency matrix A(G)

[1,]	.	1	1	1	.	.	.	.	.
[2,]	1	.	1	1	.	.	.	.	.
[3,]	1	1	.	1	.	.	.	.	.
[4,]	1	1	1	.	1	1	1	.	.
[5,]	.	.	.	1	.	1	.	.	.
[6,]	.	.	.	1	1	.	.	.	.
[7,]	.	.	.	1	.	.	.	1	1
[8,]	.	.	.	.	.	.	1	.	1
[9,]	.	.	.	.	.	.	1	1	.

Step 2: Get the degree matrix D(G)

	[,1]	[,2]	[,3]	[,4]	[,5]	[,6]	[,7]	[,8]	[,9]
[1,]	3	0	0	0	0	0	0	0	0
[2,]	0	3	0	0	0	0	0	0	0
[3,]	0	0	3	0	0	0	0	0	0
[4,]	0	0	0	6	0	0	0	0	0
[5,]	0	0	0	0	2	0	0	0	0
[6,]	0	0	0	0	0	2	0	0	0
[7,]	0	0	0	0	0	0	3	0	0
[8,]	0	0	0	0	0	0	0	2	0
[9,]	0	0	0	0	0	0	0	0	2

Step 3: Using D(G) and A(G) to obtain the Laplacian matrix L(G): L(G)=D(G)-A(G)

```
[1,]  3 -1 -1 -1  .  .  .  .  .
[2,] -1  3 -1 -1  .  .  .  .  .
[3,] -1 -1  3 -1  .  .  .  .  .
[4,] -1 -1 -1  6 -1 -1 -1  .  .
[5,]  .  .  . -1  2 -1  .  .  .
[6,]  .  .  . -1 -1  2  .  .  .
[7,]  .  .  . -1  .  .  3 -1 -1
[8,]  .  .  .  .  .  . -1  2 -1
[9,]  .  .  .  .  .  . -1 -1  2
```

This is a symmetric matrix with nine rows and nine columns.

Step 4: Compute the eigenvalues and eigenvectors of L(G)

```
eigen() decomposition
$values
[1] 7.087406e+00 4.000000e+00 4.000000e+00 3.555434e+00 3.000000e+00 3.000000e+00 1.000000e+00 3.571598e-01 2.424344e-16

$vectors
            [,1]          [,2]          [,3]        [,4]          [,5]          [,6]          [,7]       [,8]       [,9]
 [1,] -0.14936892  8.164966e-01  0.000000e+00  0.07521468  0.000000e+00  0.000000e+00  3.651484e-01 -0.2468209 -0.3333333
 [2,] -0.14936892 -4.082483e-01 -7.071068e-01  0.07521468  3.428709e-16 -7.155789e-16  3.651484e-01 -0.2468209 -0.3333333
 [3,] -0.14936892 -4.082483e-01  7.071068e-01  0.07521468 -2.788667e-16  7.795575e-16  3.651484e-01 -0.2468209 -0.3333333
 [4,]  0.90926930  4.996004e-16  0.000000e+00 -0.19220616 -6.400426e-17 -6.397867e-17  1.665335e-16 -0.1586664 -0.3333333
 [5,] -0.14936892 -1.665335e-16 -9.992007e-16  0.07521468 -2.717951e-01  6.527843e-01 -5.477226e-01 -0.2468209 -0.3333333
 [6,] -0.14936892 -1.387779e-16  8.326673e-16  0.07521468  2.717951e-01 -6.527843e-01 -5.477226e-01 -0.2468209 -0.3333333
 [7,] -0.24190035  6.383782e-16 -2.220446e-16 -0.84593401 -1.085321e-16 -4.171247e-16 -4.024558e-16  0.3387754 -0.3333333
 [8,]  0.03973784 -2.775558e-16  1.387779e-16  0.33103339 -6.527843e-01 -2.717951e-01  1.387779e-16  0.5269978 -0.3333333
 [9,]  0.03973784 -2.428613e-16  1.942890e-16  0.33103339  6.527843e-01  2.717951e-01  1.387779e-16  0.5269978 -0.3333333
```

the smallest two columns non-trivial eigenvectors the smallest two columns non-trivial eigenvectors

```
[1] -0.2468209 -0.2468209 -0.2468209 -0.1586664 -0.2468209 -0.2468209  0.3387754  0.5269978  0.5269978

[1]  3.651484e-01  3.651484e-01  3.651484e-01  1.665335e-16 -5.477226e-01 -5.477226e-01 -4.024558e-16  1.387779e-16  1.387779e-16
```

Step 5: Select the smallest two columns non-trivial eigenvectors to compute Euclidean Distance, Manhattan Distance and Angular distance, which correspond to attribute values attr1, attr2, attr3

attr1:

```
        [,1]    [,2]    [,3]    [,4]    [,5]    [,6]    [,7]    [,8]    [,9]
[1,] 0.00000 0.00000 0.00000 0.97640 2.12381 2.12381 2.16520 2.16520 2.16520
[2,] 0.00000 0.00000 0.00000 0.97640 2.12381 2.12381 2.16520 2.16520 2.16520
[3,] 0.00000 0.00000 0.00000 0.97640 2.12381 2.12381 2.16520 2.16520 2.16520
[4,] 0.97640 0.97640 0.97640 0.00000 1.14742 1.14742 0.00000 3.14159 3.14159
[5,] 2.12381 2.12381 2.12381 1.14742 0.00000 0.00000 1.99418 1.99418 1.99418
[6,] 2.12381 2.12381 2.12381 1.14742 0.00000 0.00000 1.99418 1.99418 1.99418
[7,] 2.16520 2.16520 2.16520 0.00000 1.99418 1.99418 0.00000 0.00000 0.00000
[8,] 2.16520 2.16520 2.16520 3.14159 1.99418 1.99418 0.00000 0.00000 0.00000
[9,] 2.16520 2.16520 2.16520 3.14159 1.99418 1.99418 0.00000 0.00000 0.00000
```

attr2:

```
        [,1]    [,2]    [,3]    [,4]    [,5]    [,6]    [,7]    [,8]    [,9]
[1,] 0.00000 0.00000 0.00000 0.37564 0.91287 0.91287 0.69011 0.85565 0.85565
[2,] 0.00000 0.00000 0.00000 0.37564 0.91287 0.91287 0.69011 0.85565 0.85565
[3,] 0.00000 0.00000 0.00000 0.37564 0.91287 0.91287 0.69011 0.85565 0.85565
[4,] 0.37564 0.37564 0.37564 0.00000 0.55477 0.55477 0.49744 0.68566 0.68566
[5,] 0.91287 0.91287 0.91287 0.55477 0.00000 0.00000 0.80182 0.94805 0.94805
[6,] 0.91287 0.91287 0.91287 0.55477 0.00000 0.00000 0.80182 0.94805 0.94805
[7,] 0.69011 0.69011 0.69011 0.49744 0.80182 0.80182 0.00000 0.18822 0.18822
[8,] 0.85565 0.85565 0.85565 0.68566 0.94805 0.94805 0.18822 0.00000 0.00000
[9,] 0.85565 0.85565 0.85565 0.68566 0.94805 0.94805 0.18822 0.00000 0.00000
```

attr3:

	[,1]	[,2]	[,3]	[,4]	[,5]	[,6]	[,7]	[,8]	[,9]
[1,]	0.00000	0.00000	0.00000	0.36515	0.91287	0.91287	0.58560	0.77382	0.77382
[2,]	0.00000	0.00000	0.00000	0.36515	0.91287	0.91287	0.58560	0.77382	0.77382
[3,]	0.00000	0.00000	0.00000	0.36515	0.91287	0.91287	0.58560	0.77382	0.77382
[4,]	0.36515	0.36515	0.36515	0.00000	0.54772	0.54772	0.49744	0.68566	0.68566
[5,]	0.91287	0.91287	0.91287	0.54772	0.00000	0.00000	0.58560	0.77382	0.77382
[6,]	0.91287	0.91287	0.91287	0.54772	0.00000	0.00000	0.58560	0.77382	0.77382
[7,]	0.58560	0.58560	0.58560	0.49744	0.58560	0.58560	0.00000	0.18822	0.18822
[8,]	0.77382	0.77382	0.77382	0.68566	0.77382	0.77382	0.18822	0.00000	0.00000
[9,]	0.77382	0.77382	0.77382	0.68566	0.77382	0.77382	0.18822	0.00000	0.00000

Step 6: Select the smallest three columns non-trivial eigenvectors to compute Euclidean Distance, Manhattan Distance and Angular distance, which correspond to attribute values attr4, attr5, attr6

attr4:

	[,1]	[,2]	[,3]	[,4]	[,5]	[,6]	[,7]	[,8]	[,9]
[1,]	0.00000	0.00000	0.00000	0.97640	1.93445	1.93445	2.16520	2.09176	2.09176
[2,]	0.00000	0.00000	0.00000	0.97640	1.93445	1.93445	2.16520	2.09176	2.09176
[3,]	0.00000	0.00000	0.00000	0.97640	1.93445	1.93445	2.16520	2.09176	2.09176
[4,]	0.97640	0.97640	0.97640	0.00000	1.28886	1.28886	3.14159	2.66543	2.66543
[5,]	1.93445	1.93445	1.93445	1.28886	0.00000	1.65374	1.85273	2.19511	1.48066
[6,]	1.93445	1.93445	1.93445	1.28886	1.65374	0.00000	1.85273	1.48066	2.19511
[7,]	2.16520	2.16520	2.16520	3.14159	1.85273	1.85273	0.00000	0.47616	0.47616
[8,]	2.09176	2.09176	2.09176	2.66543	2.19511	1.48066	0.47616	0.00000	0.95232
[9,]	2.09176	2.09176	2.09176	2.66543	1.48066	2.19511	0.47616	0.95232	0.00000

attr5:

	[,1]	[,2]	[,3]	[,4]	[,5]	[,6]	[,7]	[,8]	[,9]
[1,]	0.00000	0.00000	0.00000	0.38584	0.91287	0.91287	0.90509	1.15366	1.15366
[2,]	0.00000	0.00000	0.00000	0.38584	0.91287	0.91287	0.90509	1.15366	1.15366
[3,]	0.00000	0.00000	0.00000	0.38584	0.91287	0.91287	0.90509	1.15366	1.15366
[4,]	0.38584	0.38584	0.38584	0.00000	0.56173	0.56173	0.70349	0.96968	0.96968
[5,]	0.91287	0.91287	0.91287	0.56173	0.00000	0.00000	0.99290	1.22376	1.22376
[6,]	0.91287	0.91287	0.91287	0.56173	0.00000	0.00000	0.99290	1.22376	1.22376
[7,]	0.90509	0.90509	0.90509	0.70349	0.99290	0.99290	0.00000	0.26619	0.26619
[8,]	1.15366	1.15366	1.15366	0.96968	1.22376	1.22376	0.26619	0.00000	0.00000
[9,]	1.15366	1.15366	1.15366	0.96968	1.22376	1.22376	0.26619	0.00000	0.00000

attr6:

[1] 1 1 1 0 0 0 0 0 1 1 0 0 0 0 0 1 0 0 0 0 0 1 1 1 0 0 1 0 0 0 0 0 0 1 1 1

	[,1]	[,2]	[,3]	[,4]	[,5]	[,6]	[,7]	[,8]	[,9]
[1,]	0.00000	0.00000	0.00000	0.36515	0.91287	0.91287	0.58560	0.77382	0.77382
[2,]	0.00000	0.00000	0.00000	0.36515	0.91287	0.91287	0.58560	0.77382	0.77382
[3,]	0.00000	0.00000	0.00000	0.36515	0.91287	0.91287	0.58560	0.77382	0.77382
[4,]	0.36515	0.36515	0.36515	0.00000	0.65278	0.65278	0.49744	0.68566	0.68566
[5,]	0.91287	0.91287	0.91287	0.65278	0.00000	1.30557	0.65278	0.92458	0.77382
[6,]	0.91287	0.91287	0.91287	0.65278	1.30557	0.00000	0.65278	0.77382	0.92458
[7,]	0.58560	0.58560	0.58560	0.49744	0.65278	0.65278	0.00000	0.27180	0.27180
[8,]	0.77382	0.77382	0.77382	0.68566	0.92458	0.77382	0.27180	0.00000	0.54359
[9,]	0.77382	0.77382	0.77382	0.68566	0.77382	0.92458	0.27180	0.54359	0.00000

Step 7: According to the order from left to right, top to bottom, the elements of upper triangular matrix A (G) are taken, and a set of 0 and 1 sequence is obtained, the sequence is named as cla

Step 8: Combine attr1, attr2, attr3, attr4, attr5, attr6 and cla into a new data set. The attribute

variables are attr1, attr2, attr3, attr4, attr5, attr6, and the categorical variable is cla. This data set is unbalance

It is a dataset of 36 rows and 7 columns.

	A	B	C	D	E	F	G	H
1		attr1	attr2	attr3	attr4	attr5	attr6	cla
2	1	0	0	0	0	0	0	1
3	2	0	0	0	0	0	0	1
4	3	0.9764	0.37564	0.36515	0.9764	0.38584	0.36515	1
5	4	2.12381	0.91287	0.91287	1.93445	0.91287	0.91287	0
6	5	2.12381	0.91287	0.91287	1.93445	0.91287	0.91287	0
7	6	2.1652	0.69011	0.5856	2.1652	0.90509	0.5856	0
8	7	2.1652	0.85565	0.77382	2.09176	1.15366	0.77382	0
9	8	2.1652	0.85565	0.77382	2.09176	1.15366	0.77382	0
10	9	0	0	0	0	0	0	1
11	10	0.9764	0.37564	0.36515	0.9764	0.38584	0.36515	1
12	11	2.12381	0.91287	0.91287	1.93445	0.91287	0.91287	0
13	12	2.12381	0.91287	0.91287	1.93445	0.91287	0.91287	0
14	13	2.1652	0.69011	0.5856	2.1652	0.90509	0.5856	0
15	14	2.1652	0.85565	0.77382	2.09176	1.15366	0.77382	0
16	15	2.1652	0.85565	0.77382	2.09176	1.15366	0.77382	0
17	16	0.9764	0.37564	0.36515	0.9764	0.38584	0.36515	1
18	17	2.12381	0.91287	0.91287	1.93445	0.91287	0.91287	0
19	18	2.12381	0.91287	0.91287	1.93445	0.91287	0.91287	0
20	19	2.1652	0.69011	0.5856	2.1652	0.90509	0.5856	0
21	20	2.1652	0.85565	0.77382	2.09176	1.15366	0.77382	0
22	21	2.1652	0.85565	0.77382	2.09176	1.15366	0.77382	0
23	22	1.14742	0.55477	0.54772	1.28886	0.56173	0.65278	1
24	23	1.14742	0.55477	0.54772	1.28886	0.56173	0.65278	1
25	24	0	0.49744	0.49744	3.14159	0.70349	0.49744	1
26	25	3.14159	0.68566	0.68566	2.66543	0.96968	0.68566	0
27	26	3.14159	0.68566	0.68566	2.66543	0.96968	0.68566	0
28	27	0	0	0	1.65374	0	1.30557	1
29	28	1.99418	0.80182	0.5856	1.85273	0.9929	0.65278	0
30	29	1.99418	0.94805	0.77382	2.19511	1.22376	0.92458	0
31	30	1.99418	0.94805	0.77382	1.48066	1.22376	0.77382	0
32	31	1.99418	0.80182	0.5856	1.85273	0.9929	0.65278	0
33	32	1.99418	0.94805	0.77382	1.48066	1.22376	0.77382	0
34	33	1.99418	0.94805	0.77382	2.19511	1.22376	0.92458	0
35	34	0	0.18822	0.18822	0.47616	0.26619	0.2718	1
36	35	0	0.18822	0.18822	0.47616	0.26619	0.2718	1
37	36	0	0	0	0.95232	0	0.54359	1

Step 9: Use SMOTE method to get the balanced data sets

It is a dataset of 78 rows and 7 columns.

	A	B	C	D	E	F	G	H
1		attr1	attr2	attr3	attr4	attr5	attr6	cla
2	26	3.14159	0.68566	0.68566	2.66543	0.96968	0.68566	0
3	6	2.1652	0.69011	0.5856	2.1652	0.90509	0.5856	0
4	12	2.12381	0.91287	0.91287	1.93445	0.91287	0.91287	0
5	30	1.99418	0.94805	0.77382	1.48066	1.22376	0.77382	0
6	26.1	3.14159	0.68566	0.68566	2.66543	0.96968	0.68566	0
7	28	1.99418	0.80182	0.5856	1.85273	0.9929	0.65278	0
8	7	2.1652	0.85565	0.77382	2.09176	1.15366	0.77382	0
9	12.1	2.12381	0.91287	0.91287	1.93445	0.91287	0.91287	0
10	6.1	2.1652	0.69011	0.5856	2.1652	0.90509	0.5856	0
11	30.1	1.99418	0.94805	0.77382	1.48066	1.22376	0.77382	0
12	13	2.1652	0.69011	0.5856	2.1652	0.90509	0.5856	0
13	30.2	1.99418	0.94805	0.77382	1.48066	1.22376	0.77382	0
14	13.1	2.1652	0.69011	0.5856	2.1652	0.90509	0.5856	0
15	21	2.1652	0.85565	0.77382	2.09176	1.15366	0.77382	0
16	18	2.12381	0.91287	0.91287	1.93445	0.91287	0.91287	0
17	12.2	2.12381	0.91287	0.91287	1.93445	0.91287	0.91287	0
18	15	2.1652	0.85565	0.77382	2.09176	1.15366	0.77382	0
19	5	2.12381	0.91287	0.91287	1.93445	0.91287	0.91287	0
20	7.1	2.1652	0.85565	0.77382	2.09176	1.15366	0.77382	0
21	5.1	2.12381	0.91287	0.91287	1.93445	0.91287	0.91287	0
22	26.2	3.14159	0.68566	0.68566	2.66543	0.96968	0.68566	0
23	30.3	1.99418	0.94805	0.77382	1.48066	1.22376	0.77382	0
24	15.1	2.1652	0.85565	0.77382	2.09176	1.15366	0.77382	0
25	12.3	2.12381	0.91287	0.91287	1.93445	0.91287	0.91287	0
26	15.2	2.1652	0.85565	0.77382	2.09176	1.15366	0.77382	0
27	17	2.12381	0.91287	0.91287	1.93445	0.91287	0.91287	0
28	25	3.14159	0.68566	0.68566	2.66543	0.96968	0.68566	0
29	29	1.99418	0.94805	0.77382	2.19511	1.22376	0.92458	0
30	20	2.1652	0.85565	0.77382	2.09176	1.15366	0.77382	0
31	21.1	2.1652	0.85565	0.77382	2.09176	1.15366	0.77382	0
32	7.2	2.1652	0.85565	0.77382	2.09176	1.15366	0.77382	0
33	20.1	2.1652	0.85565	0.77382	2.09176	1.15366	0.77382	0
34	14	2.1652	0.85565	0.77382	2.09176	1.15366	0.77382	0
35	13.2	2.1652	0.69011	0.5856	2.1652	0.90509	0.5856	0
36	26.3	3.14159	0.68566	0.68566	2.66543	0.96968	0.68566	0
37	15.3	2.1652	0.85565	0.77382	2.09176	1.15366	0.77382	0
38	21.2	2.1652	0.85565	0.77382	2.09176	1.15366	0.77382	0
39	4	2.12381	0.91287	0.91287	1.93445	0.91287	0.91287	0
40	4.1	2.12381	0.91287	0.91287	1.93445	0.91287	0.91287	0
41	1	0	0	0	0	0	0	1

42	2	0	0	0	0	0	0	1
43	3	0.9764	0.37564	0.36515	0.9764	0.38584	0.36515	1
44	9	0	0	0	0	0	0	1
45	10	0.9764	0.37564	0.36515	0.9764	0.38584	0.36515	1
46	16	0.9764	0.37564	0.36515	0.9764	0.38584	0.36515	1
47	22	1.14742	0.55477	0.54772	1.28886	0.56173	0.65278	1
48	23	1.14742	0.55477	0.54772	1.28886	0.56173	0.65278	1
49	24	0	0.49744	0.49744	3.14159	0.70349	0.49744	1
50	27	0	0	0	1.65374	0	1.30557	1
51	34	0	0.18822	0.18822	0.47616	0.26619	0.2718	1
52	35	0	0.18822	0.18822	0.47616	0.26619	0.2718	1
53	36	0	0	0	0.95232	0	0.54359	1
54	110	0	0.010049197	0.010049197	0.025422514	0.014212069	0.014511591	1
55	210	0	0	0	0	0	0	1
56	31	0	0	0	0.313896197	0	0.179173842	1
57	41	0	0	0	0	0	0	1
58	51	1.099165522	0.504227229	0.496206609	1.200697246	0.512101418	0.571623202	1
59	61	1.058839872	0.461989282	0.453157528	1.12702076	0.470627446	0.503801505	1
60	71	0	0	0	0.16338192	0	0.093259385	1
61	8	0	0	0	0	0	0	1
62	91	1.138023966	0.544928394	0.537689396	1.271693092	0.552066403	0.63697728	1
63	101	0.932737166	0.367258918	0.357238012	0.954030176	0.380489469	0.360975558	1
64	11	1.046301336	0.448856152	0.43977219	1.104112382	0.457731861	0.482713567	1
65	121	0.969393017	0.374295009	0.363880289	0.972810105	0.38498135	0.364480088	1
66	131	1.073353389	0.47719105	0.468651229	1.153537504	0.485554253	0.528211064	1
67	141	1.031255388	0.433096705	0.423710099	1.076622866	0.442257461	0.457408538	1
68	151	0.541534634	0.524497381	0.521170074	2.267178308	0.636585162	0.570754035	1
69	161	1.14742	0.55477	0.54772	1.28886	0.56173	0.65278	1
70	171	0.383282161	0.516590412	0.514235443	2.522707326	0.656136739	0.549329501	1
71	181	0.012525537	0.495877515	0.495742946	3.113814326	0.699415095	0.495742946	1
72	19	0	0	0	1.010853881	0	0.607177645	1
73	201	0	0.078445114	0.078445114	1.162955826	0.110940946	0.874722027	1
74	211	0	0.00562532	0.00562532	0.014230965	0.007955604	0.00812327	1
75	221	0	0.005479613	0.005479613	0.013862355	0.007749538	0.007912861	1
76	231	0	0.166225357	0.166225357	0.420517831	0.235084092	0.24003853	1
77	241	0	0.18822	0.18822	0.47616	0.26619	0.2718	1
78	251	0	0.150591481	0.150591481	0.571352836	0.212973894	0.326135645	1
79	261	0	0	0	0.584962129	0	0.333899911	1

Step 10： Use RF for the classification prediction on the data sets obtained in Step 9
This is the entire step of the LPbSA algorithm.
